# Effects of visitor pressure on understory vegetation in Warsaw forested parks (Poland)

**DOI:** 10.1007/s10661-012-2987-0

**Published:** 2012-11-11

**Authors:** Piotr Sikorski, Iwona Szumacher, Daria Sikorska, Marcin Kozak, Marek Wierzba

**Affiliations:** 1 Department of Environmental Protection, Warsaw University of Life Sciences—SGGW, 159 Nowoursynowska Str., 02-776 Warsaw, Poland; 2 Department of Geoecology, University of Warsaw, 30 Krakowskie Przedmieście Str., 00-927 Warsaw, Poland; 3 Department of Environmental Improvement, Warsaw University of Life Sciences—SGGW, 159 Nowoursynowska Str., 02-776 Warsaw, Poland; 4 Department of Botany, Faculty of Agriculture and Biology, Warsaw University of Life Sciences—SGGW, 159 Nowoursynowska Str., 02-776 Warsaw, Poland; 5 Department of Botany, Institute of Biology, Siedlce University of Natural Sciences and Humanities, 3 Maja Str., 08-110 Siedlce, Poland

**Keywords:** Trampling, Understorey vegetation, Urban park, Ancient forest, Homogenisation

## Abstract

Visitor’s access to understorey vegetation in park forest stands results in the impoverishment of plant species composition and a reduction in habitat quality. The phenomenon of biotic homogenisation is typical in urban landscapes, but it can proceed differently depending on the scale, a detail that has not been observed in previous studies. This research was carried out in seven Warsaw parks (both public and restricted access). Thirty-four forested areas were randomly selected, some subjected to strong visitors’ pressure and some within restricted access areas, free of such impacts. The latter category included woodlands growing in old forest and secondary habitats. Public access to the study areas contributed to the disappearance of some forest species and their replacement by cosmopolitan non-forest species, leading to loss of floristic biodiversity in areas of high ecological importance at the city scale. Some human-induced factors, including soil compaction and changes in soil pH, moisture and capillary volume, were found to cause habitat changes that favoured native non-forest plants. Despite changes in species composition, the taxonomic similarity of understorey vegetation in both categories—public access and restricted access—was comparable. In a distance gradient of measurements taken around selected individual trees, there was found to be significant variation (in light, soil pH and compaction) affecting the quality and quantity of understorey vegetation (including rare species). In conclusion, the protection of rare forest species could be achieved by limiting access to forested areas, particularly in old forest fragments, and we highly recommend its consideration in the proposal of future park restoration plans.

## Introduction

One of the important activities counteracting the impoverishment of biodiversity in towns is the protection of natural resources, some of which are located in urban parks (Celesti-Grapow et al. 2006). Many studies have been undertaken to increase our understanding of factors that facilitate a high diversity of native species in urban parks (Hermy and Cornelis 2000; Nakamura et al. 2005; Weifeng et al. 2006; LaPaixa and Freedman 2010). Trampling (Jim 1998) and other factors associated with the presence of users, referred to as “visitor pressure” (Sarah and Zhevelev 2007; Zhevelev and Sarah 2008), are assumed to be the most important reasons for the decline in species diversity of understory vegetation in parks. Negative effects of trampling on the species diversity of understory plants have been reported for urban habitats (Bhuju and Ohsawa 1998; Sarah and Zhevelev 2007; Zhevelev and Sarah 2008). Urban forest stands are usually spatially isolated ecosystems, so these fragments play a key role in biodiversity conservation (Hermy 1994; Hermy et al. 1999). However, there have been no comparative studies showing how the species composition of the vegetation in trampled parks is simplified with respect to ancient and recent forest stands. Little is known about the effects of water deficits on understory vegetation, and this is of particular importance for urban heat islands and for maintaining biodiversity in urban green spaces (Wenga et al. 2004). Understanding these factors is important for the identification and management of park types with the aim of maintaining plant diversity and for determining habitat factors responsible for homogenisation. Due to the high mobility of park users and the resulting mosaic character of understory destruction, it is important to understand this phenomenon both at the ecosystem scale and micro-environments (Sarah and Zhevelev 2007; Zhevelev and Sarah 2008).

The aim of this study was to test the hypothesis that understory vegetation in park forest stands subjected to visitor pressure underwent homogenisation in various tree stands and in microhabitats. Differences in plant composition and in some substrate parameters were determined between forest stands subjected to visitor pressure and those free from such impact. The habitat characteristics most affecting the differentiation of vegetation were assessed.

## Methods

### Study area

**Fig. 1:**
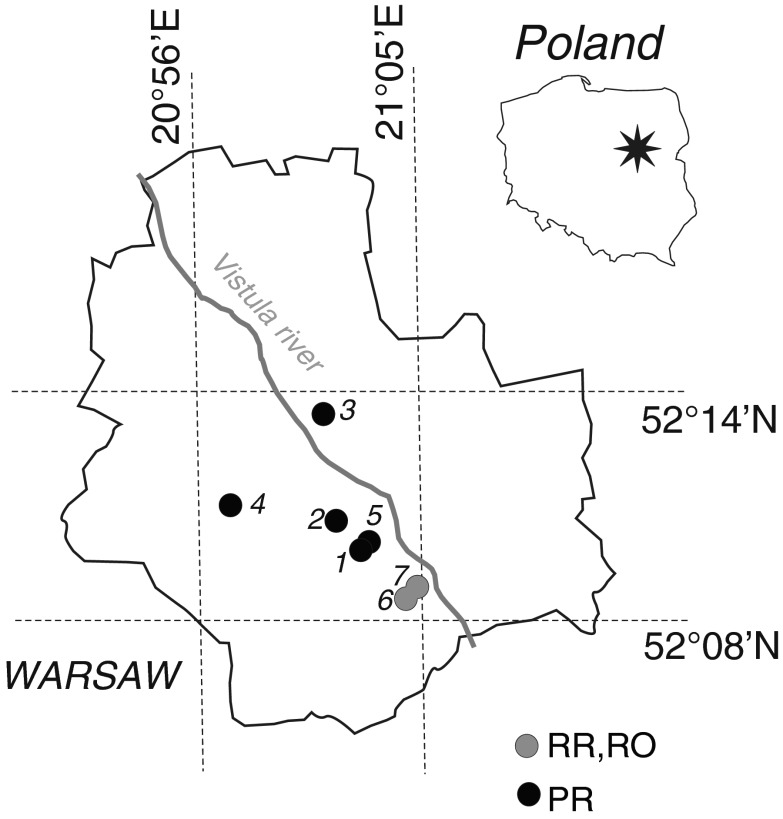
Location of the studied urban parks within city limits with public access to undergrowth (*PR*) and restricted access to undergrowth vegetation (*RR* and *RO*), the numbers of the objects are as in Table 1

The effect of visitor pressure on habitats and micro-habitats was analysed at seven urban parks in Warsaw, where forest stand areas were studied (Fig. 1). The investigated area is situated in the Środkowomazowiecka Plain, which is a region characterised by a transitory oceanic-continental eastern European climate with typically high annual variability of weather conditions. As an urban heat island, Warsaw has a specific climate which is different from the one in surrounding areas, as it exhibits higher air temperatures (Lorenc and Mazur 2003). The mean annual precipitation was 595 mm, and there was a relatively high mean annual temperature of 9.3 °C, i.e. values close to long-term averages (data from the meteorological station in Warsaw-Okęcie 2008–2009). Vegetation of the investigated parks consists of forest stands, grasslands and sporadically (less than 0.1 %) other areas, for example shrub plantations, rose gardens and short-lived ruderal communities. Forest stands (units composed of more or less natural forest vegetation in the sense of Hermy and Cornelis (2000)) always covered more than half of the whole park area. The intensity of human impacts in the parks is variable. All of the parks are situated near large housing estates and are frequently visited by their residents (Table 1).

**Table 1:** Characteristics of the studied urban parks and physical, chemical soil properties of in the selected forest stands (values averaged for analysed sites)

Park ID	Park name	Established	Area (ha)	% forest cover	Access to undergrowth	Forest stand continuity in park^a^	Forest stand type^b^	Number of analysed forest stand/sample plots	Humus (%)	Compaction (N/cm^2^)	Sand in %	Silt in %	Clay in %
(1)	Arkadia Górna	1973	6.4	72	Yes (P)	Recent (R)	PR	2/24	1.80	362	67	31	2
(2)	Morskie Oko (Dolne)	1774	17.9	63	Yes (P)	Recent (R)	PR	7/84	4.32	269	60	38	2
(3)	Skaryszewski	1906	49.6	62	Yes (P)	Recent (R)	PR	2/24	3.70	289	23	76	1
(4)	Sowińskiego	1936	8.3	73	Yes (P)	Recent (R)	PR	2/24	4.25	344	60	38	2
(5)	Arkadia Dolna	1973	9.7	54	Yes (P)	Recent (R)	PR	3/36	3.30	185	–	–	–
(6)	Park Natolin (Górny)	1780	26.0	75	No (R)	Recent (R)/old (O)	RR/RO	2/24	2.45	162	81	18	1
(7)	Park Natolin (Dolny)	1780	79.0	98	No (R)	Recent (R)/old (O)	RR/RO	16/192	3.72	173	74	25	1

^a^Recent (R)—ca 60 years old, old (O)—over 150 years

^b^RO—old (ancient) forest stand with restricted access to undergrowth vegetation, RR—recent forest stand with restricted access to undergrowth vegetation, PR—recent forest stand with public access

### Data collected

Due to varying access to the understorey, the forest stands located within urban parks were divided into restricted access areas (R; limited access and an over 60-year-long ban on walking out of the park’s trails) and public access areas (P; visited regularly for over 40 years with unlimited access to the understorey), where the number of visitors in single forest stand was estimated to be between 7 and 35 per week during summer. Depending on habitat persistence, the forest stands were divided into old (ancient; O) forest stands, which have maintained habitat continuity for over 150 years, and recent forest stands (R), which were planted approximately 60 years ago on non-forest grounds (based on aerial photos from the years 1945–2004, Lindley’s plans from the years 1883–1915 at scales of 1:500 to 1:1,000 and a plan of the park in Natolin from 1,859 stored in the State Archives of Old Acts in Warsaw; Table 1). Moreover, three types of forest stands at the age of 60 years, dominated by *Acer pseudoplatanus*, *Acer platanoides* or *Tilia cordata* commonly found in Warsaw parks were singled out. In every forest stand type (PR, RR, RO) even number of sample plots was randomly preselected for further verification if they consist of the tree species mentioned above of an age about 60 years. Throughout 2008 and 2009 in each of the 34 forest stands that fulfilled the criteria, transects emanating from trunks of randomly selected trees and running parallel to the cardinal compass points were created. The number of trees depended on the size of the forest stand. Along each transect, beginning 1 m from the base of a trunk, three 1 × 1 m quadrates were sampled at points 1.5 m apart. Vegetation composition was recorded, soil samples of 100 g volume collected, and other habitat parameters measured. Organic matter content was determined using the titration procedure. For particle size distributions between 0.01 and 2,000 μm, the laser diffraction method was applied. Selected soil properties in the upper soil layer (0–10 cm) were, according to Bhuju and Ohsawa (1998), Jim (1998), Haase et al. (2000), Sarah and Zhevelev (2007) and Zhevelev and Sarah (2008), important factors affecting plant composition. The results of forest stand availability to off-trail visits are changes in the habitat and their effect on understorey vegetation. Mechanical damage to plants was ignored as the plants have remained under constant pressure and species sensitive to trampling have already receded. Vascular plant species abundance, their height and coverage were assessed at the end of July and beginning of August. Earlier, in May, the list of spring geophytes (Kühn et al. 2004) was performed. The names of vascular plant species and of alien species were recorded following Mirek et al. (2002), forest and non-forest species were adopted after Matuszkiewicz (2008; appendix 1), ancient forest species by Dzwonko (2007; Appendix 1). Plant percentage cover was assessed approximately by visual evaluation in the field. The Shannon index was used as a measure of biological diversity (Magurran 2004). The average height of the understorey was calculated as the mean of five random measurements. Soil compaction was analysed 3 days after the last rainfall with piston penetrometer Eijkelkamp 06.01.SA. The resistance of the probe was measured in at least 10 points evenly distributed over the area. The amount of light was measured with a DataLogger LI-1400 equipped with a Quantum Sensor LI-190SA 0.1 m above the ground surface and above the understory canopy at a height of 1 m according to Messier and Puttonen (1995). Moisture, temperature and salinity were measured with a W.E.T. (HH2) probe in soil at a depth of 0–10 cm and the pH was measured with CPC-502 instrument. Changes in moisture and temperature between water saturated and dry soil were assessed from the difference in the means for these states. Measurements were made on 3 days: at a temperature of 20–25 °C in midday, a day after torrential rains (at least 20 mm) and in a dry state, 3 days after rainfall. Bulk density, moist bulk density and capillary water capacity were measured using cylinders with a capacity of 100 cm^3^. The sampled material was weighed fresh in a dry state (dried for 24 h at 105 °C) and in a state of capillary saturation.

### Statistical analysis

The influences of the forest stand type (PR, RR and RO), gradient of a single tree crown, direction and their interactions were analysed using linear mixed effects models (Pinheiro and Bates 2000). Multiple comparisons for linear and generalized linear mixed effects models were employed (Hothorn et al. 2008) with no adjustment for multiple testing (Webster 2007; Kozak 2009). The mixed effects framework was used because of the nesting of observations within samples being taken close to a single tree; the trees were nested within parks. The fitted models were checked graphically and, in case of problems with the residual distribution, the variance–covariance structure was modelled using a power function, which worked well in all cases (Pinheiro and Bates 2000). For count data (native species, native forest species, alien species, spring geophytes and ancient forest species), generalized linear mixed effects models with the Poisson distribution were estimated through penalized quasi-likelihood (Venables and Ripley 2002). The significance level for all analyses was 0.05. The computations were performed in the nlme package of R (R Development Core Team 2010). Log-transformed species abundances and environmental variables were implemented into multivariate CCA analysis. The significance of the five most important factors was based on a Monte Carlo algorithm. Multivariate analysis was performed with the CANOCO 4.5 programme (ter Braak and Šmilauer 2002).

## Results

### Vegetation and environmental conditions in different forest stand types

**Fig. 2:**
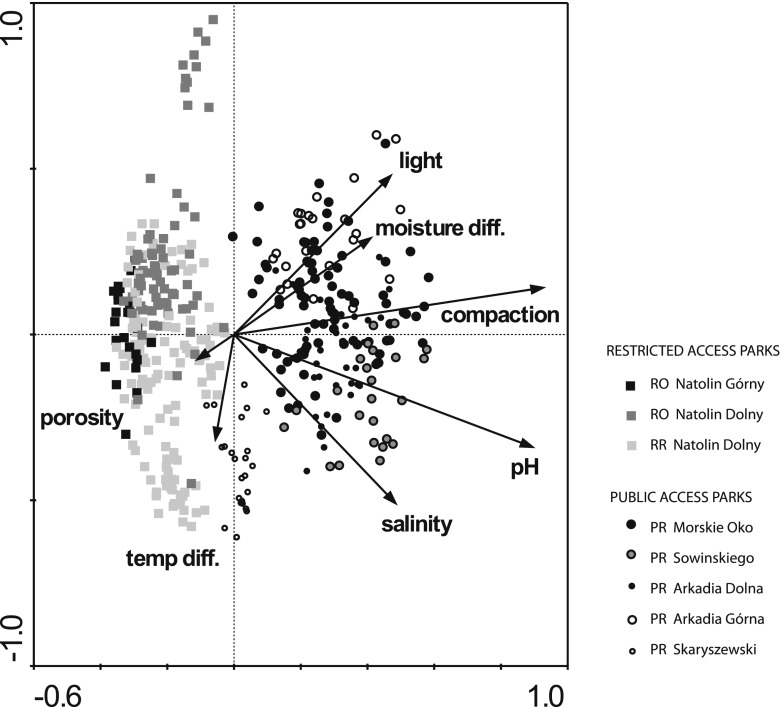
CCA diagram for all samples with habitat variables; *light*—light 1 m above ground, *moisture diff.*—moisture differences in hot days, *compaction*—soil compaction at a depth of 5 cm, *pH*_*H2O*_ *, salinity, temperature diff.*—temperature differences in hot days

**Fig. 3:**
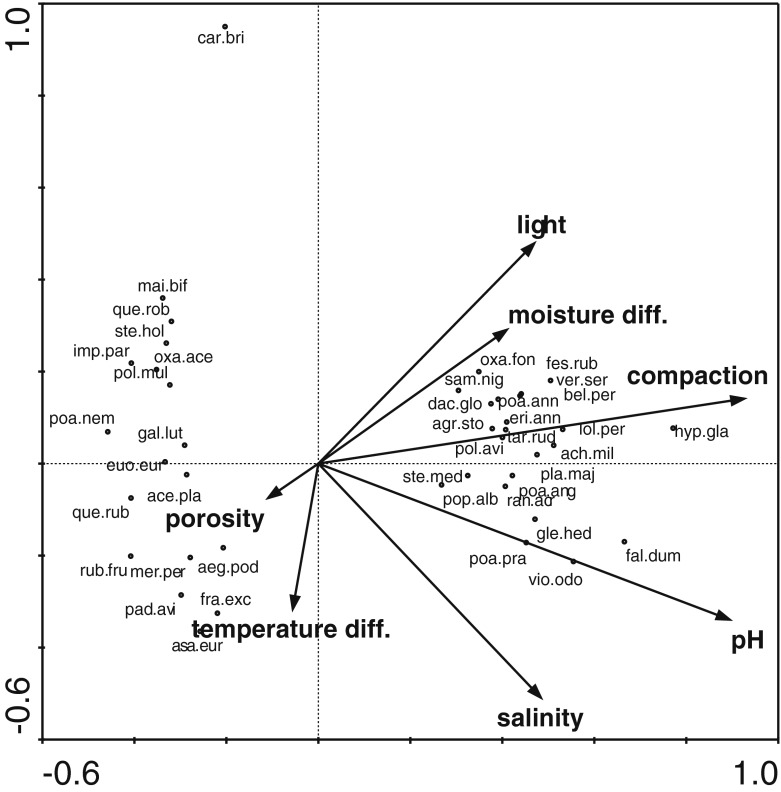
CCA diagram for plant species and habitat variables, plant names are presented in Appendix 1

Forest stands subjected to pressure from park visitors (PR) had a lower number of all species and of native species in comparison to those located in restricted access areas (RR, RO; Table 2). The most remarkable difference in species richness was recorded for forest species, whose number was over 30 times higher in RR stands (Table 2). The understorey vegetation of PR stands was characterised by reduced height but its coverage was similar to RR (Table 2). No differences in the number of native non-forest species, geophytes and ancient forest species were observed between PR and RR forest stands; however, the percentage of ancient forest species was different from that of native forest species (Table 2). The habitat parameters in both types of forest stands were similar, small differences were found only in moist bulk density (Table 2). The habitats of PR forest stands exhibited many features hampering the growth of understorey plants: higher pH, soil compaction, insolation, bulk density and moisture variability (Table 2). Overall the CCA model explained 32.6 % of variance in species abundance with the highest contribution of soil compaction, pH, salinity, light intensity, moisture differentiation, temperature difference and porosity. The understorey vegetation clearly differentiated into two groups (Fig. 2). The first group was associated with restricted access areas and consisted of such species as: *Aegopodium podagraria*, *Asarum europaeum*, *Galeobdolon luteum*, *Maianthemum bifolium*, *Mercurialis perennis*, *Polygonum multiflorum* and *Stellaria holostea*. The second group is represented by ruderal and native non-forest species attached to public access areas such as: *Achillea Millefolium*, *Agrostis stolonifera*, *Glechoma hederacea*, *Poa annua*, *Poa angustifolia*, *Stellaria media* or *Veronica serpyllifolia* (Fig. 3).

**Table 2:** Mean values of understorey vegetation and soil characteristics in different forested areas, different letters in the row indicate a significant difference between the two corresponding means, significant differences at *p* < 0.05, according to the asymptotic *z* test used for multiple comparisons in linear and generalized linear mixed effects models, different letters in a row indicate a significant difference between the two corresponding means

	PR	RR	RO
Vegetation characteristics			
Species richness (*n*)	5.79 a	8.03 b	8.17 b
Native species (*n*)	5.44 a	7.88 b	7.84 b
Native non-forest species (%)	40.32 a	8.30 b	0.91 b
Native forest species (%)	1.40 a	46.9 b	58.5 c
Alien species (*n*)	0.35 a	0.14 a	0.32 a
Ancient forest species (*n*)	1.16 a	6.19 b	6.46 b
Spring geophytes (*n*)	0.99 a	3.42 b	3.57 b
Diversity index	1.13 a	1.16 a	1.18 a
Plant height (cm)	13.65 a	31.23 b	31.82 b
Plant cover (%)	42.94 a	55.45 b	59.84 b
Soil characteristics			
Salinity (mS/cm)	0.83 a	0.67 ab	0.59 b
pH (H_2_O)	7.01 a	5.28 b	4.77 b
Temperature difference (°C)	3.36 a	5.24 a	3.17 a
Soil compaction (N/cm^2^)	323 a	76 b	89 b
Light 0.1 m above ground (%)	14.03 a	2.85 b	4.37 b
Light 1 m above ground (%)	14.10 a	2.93 b	10.26 ab
Bulk density (g·cm^−3^)	1.22 a	1.03 b	0.96 b
Moist bulk density (g·cm^−3^)	1.46 a	1.27 b	1.13 c
Difference in moisture (%)	12.52 a	5.92 b	8.70 ab
Capillary water capacity (% volume)	35.90 a	36.60 a	–
Capillary water capacity (% weight)	29.80 a	36.10 b	–

The effect of dominant tree species in the canopy was non-significant with respect to habitat conditions (Table 4; the effect of tree species). However, it affected the native plant coverage and number of species in the understorey (Table 3).

**Table 3:** The effect of forest stand type, gradient and direction and their second-order interactions on the mean number and percentage cover of plant species and their diversity (values significant at *p* < 0.05 are presented in bold)

	Floristic richness (*n*)	Native species (*n*)	Native forest species (%)	Native non-forest species (%)	Alien species (*n*)	Spring geophytes (*n*)	Ancient forest species (*n*)	Diversity index	Plant height (cm)	Plant cover (%)
Type	**<0.001**	**<0.001**	**<0.001**	**<0.001**	0.060	**<0.001**	**<0.001**	0.837	**<0.001**	**0.006**
Gradient	**<0.001**	**<0.001**	**<0.001**	**<0.001**	0.216	0.677	**0.006**	**0.005**	**<0.001**	**<0.001**
Direction	**0.005**	**0.001**	**0.002**	**0.007**	0.092	0.604	0.189	0.467	0.363	0.485
Tree species	0.100	**0.030**	**0.013**	**0.010**	0.063	0.194	0.170	0.507	0.389	**0.003**
Type × gradient	**0.003**	**<0.001**	**<0.001**	**<0.001**	0.098	0.312	**0.002**	0.096	**<0.001**	**0.047**
Type × direction	**0.030**	**0.006**	**<0.001**	0.462	**0.016**	0.110	0.282	0.625	0.386	**0.001**
Gradient × direction	0.151	**0.047**	**0.037**	0.821	0.100	0.281	**0.039**	0.831	0.851	0.608
Gradient × tree species	**0.036**	**0.014**	**0.018**	**<0.001**	0.076	0.280	0.095	0.064	0.175	**0.002**
Direction × tree species	0.117	**0.037**	**0.034**	0.275	0.066	**0.019**	**0.006**	0.082	0.566	0.878

### Microhabitat conditions in the gradient of single tree crowns

**Fig. 4:**
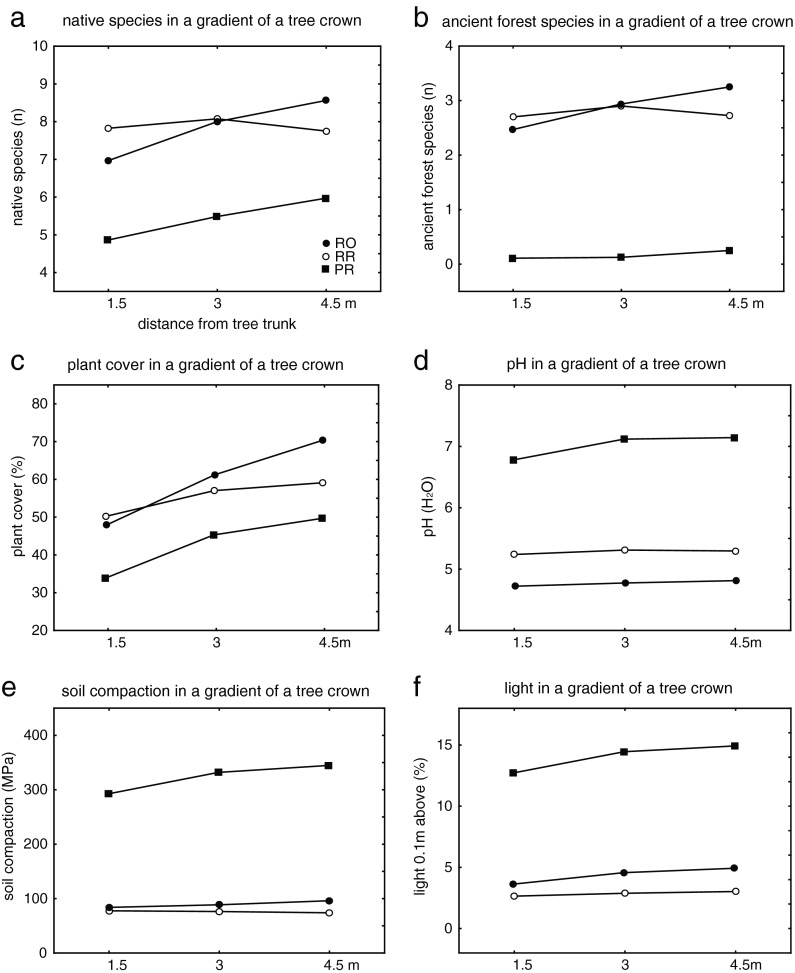
Differentiation of selected vegetation and habitat conditions in the gradient of single tree crowns

The indicators of species composition, e.g. floristic richness, number of native species and their diversity, and indicators of understorey vegetation structure, e.g. plant height and plant cover, change when approaching the tree trunk (Table 3). Among the environmental factors analysed, only pH, temperature difference, light and soil bulk density differed significantly along the gradient of single tree crowns (Table 4). The level of the decrease of the above factors differed among forest stands (Fig. 4). In PR forest type, in contrast to RO and RR, the decrease was noticeable for each of the environmental factors (Fig. 4). It was unexpected, in the context of habitats homogenisation in the cities (Sarah and Zhevelev 2007), that environmental factors, differentiated with the increasing distance from the tree in PR forest stands.

**Table 4:** The effects of woodland type, gradient and direction and their second-order interactions on soil characteristics (values significant at *p* < 0.05 are presented in bold)

	Salinity (mS/cm)	pH (H_2_O)	Temperature difference (°C)	Soil compaction (MPa)	Light 0.1 m above (%)	Light 1 m above (%)	Bulk density (g·cm^−3^)	Capillary water capacity (% volume)	Capillary water capacity (% weight)	Difference in moisture content (%)
Type	**<0.001**	**<0.001**	0.287	**<0.001**	**<0.001**	**0.029**	**<0.001**	0.704	**0.005**	**0.010**
Gradient	0.708	**<0.001**	**<0.001**	0.943	**<0.001**	**<0.001**	**0.001**	0.700	0.107	0.548
Direction	0.174	0.643	**<0.001**	**0.048**	**<0.001**	**0.031**	0.927	0.387	0.365	0.104
Tree species	0.235	0.553	0.796	0.396	0.524	0.833	0.804	0.858	0.936	0.557
Type × gradient	0.059	**0.010**	0.980	**0.002**	**<0.001**	**0.023**	0.330	0.067	**0.015**	0.636
Type × direction	**0.042**	0.391	0.050	**0.006**	**<0.001**	**0.002**	0.409	0.460	0.441	0.125
Gradient × direction	0.997	0.923	**0.003**	0.792	0.847	0.845	0.748	0.458	0.256	0.473
Gradient × tree species	0.150	0.444	0.322	0.737	**<0.001**	0.415	0.437	0.638	0.315	0.254
Direction × tree species	0.866	**0.014**	0.929	0.773	**<0.001**	**0.003**	0.536	0.308	0.359	0.696

Plant cover and species composition along the gradient were determined by the tree species dominating in the canopy (Table 3; gradient × tree interaction). No effect of tree species and the gradient on habitat conditions was detected (Table 4; interaction gradient × tree).

## Discussion

### Homogenisation of understorey vegetation in various tree stands

Biotic homogenisation is the process of increasing the uniformity of local habitats due to replacement of native species by alien species and is associated with increased taxonomic similarity (McKinney and Lockwood 1999; McKinney 2006; Olden and Rooney 2006). This concept explains the complex phenomenon of the formation of similar habitats as an effect of various human activities (Savard et al. 2000; Schwartz et al. 2006). However, Olden et al. (2004) indicated that this idea may not be sufficient to unequivocally explain processes taking place at various spatial and temporal scales thus homogenisation may proceed simultaneously to differentiation. In urban forest stands the understorey vegetation under pressure undergoes homogenisation, but differentiation within some environmental variables occurs as well (Fig. 4).

Visitor pressure in the studied forest stands contributed to declines in the multi-species composition of forest plants, a decrease in plant height and cover, and their replacement by non-forest plants. Previously, Gomez-Limon and de Lucio (1995), Sarah and Zhevelev (2007), Zhevelev and Sarah (2008) and Hamberg et al. (2010) reported fewer species and an impoverished structure of lower percentage cover and height in areas associated with visitor pressure. Floristic impoverishment pertains mainly to the group of forest plants including ancient forest species and geophytes, which are typical elements for natural forest communities in fertile habitats of oak–lime–hornbeam forests (Matuszkiewicz 2008). Forest stands in urban parks and forests, as a refuge for these species in urbanised environments, lose their taxonomic identity under the impact of park users (DeCandido 2004; Amrein et al. 2005). Maintenance of the pool of forest species and their habitats is difficult because such forest stands, even if not affected by park users, are generally established on non-forest grounds, and hence, are associated with limited opportunities for the preservation of forest species of low dispersion ability (Hermy et al. 1999). This fact explains differences found in the number of species of RO and RR forest stands (Table 2). The period of effective colonisation is assumed to be 200 years for ancient forests and 100 years for old forests (Hermy et al. 1999; Wulf 2003). Habitat continuity of 60 years is insufficient to support most ancient forest species. A decline in the number of geophytes and forest species was also observed in RO type. Retreating species are replaced by species associated with open areas, facilitated by a looser structure of tree stands that manifest in greater insulation at ground level (Table 2). Such a structure is an effect of trampling and selective care when removing undergrowth, which consequently results in a smaller distances among young trees. In turn, this smaller closure affects the density of lower canopy layers and is important for light penetration to a height of approximately 1 m above the ground (Lhotka and Loewenstein 2006). The influence of stand dominant direction was related to light (Table 3), which indicated the effect of forest edges in the tree canopy and hence, a higher possibility of penetration by non-forest species (Hamberg et al. 2008; Hamberg et al. 2010). The share of alien species was small in the understory of the studied forest stands; such species usually appear only in association with heavily trampled trails (Nepal and Wayb 2007). The number of new non-forest species in PR forest stands subjected to visitor pressure was sufficiently high to affect plant floristic diversity and was higher than the numbers found in RO and RR forest stands. For forest species that are sporadically present in urban environments (sometimes called “the losers”), this phenomenon is unfavourable and contributes to homogenisation (McKinney and Lockwood 1999; Ricotta et al. 2008).

### Effect of users’ pressure on park habitat conditions and its effect on vegetation

Changes in understorey vegetation affected by users’ pressure result from changes in the habitat, which loses the properties typical of soils from stable forest ecosystems. Direct effects of such changes are the compaction of mineral soil particles and increased soil bulk density (Bhuju and Ohsawa 1998; Kozlowski 1999). In the case of urban woodlands, the differences in bulk density and soil compaction between RO and RR forest stands were insignificant suggesting that the cycling of organic matter were irrespective of age (Table 2). Furthermore, as reported by Scharenbroch et al. (2005), for example, the younger the soil in an urban ecosystem, the higher its bulk density. Also, microbial activity, which plays an essential role in organic matter decomposition and nutrient cycling, increases significantly in areas with less human trampling or without recreational activity (Lucas-Borja et al. 2011). However, Kissling et al. (2009) suggested that microbial activity is difficult to predict from a long-term perspective. The typical bulk density of intact mineral soils (Bullock and Gregory 1991) is similar to that noted in the studied park forest stands, which ranges between 0.96 and 1.22 g·cm^−3^ (Table 2). Critical values exceeding 1.3 g·cm^−3^ and accompanying declines of plant cover were noted in soils of forest trails (Chappell et al. 1971; Bhuju and Ohsawa 1998; McDonald 2008). High soil compaction in parks may be caused by regular movement of the equipment used for park maintenance, which can result in a soil bulk density change from 1.38 to 1.53 g·cm^−3^ as reported by Garten et al. (2003). Bhuju and Ohsawa (1998) found significant relationships between soil density and understory plant coverage, but no such relationships were noted in the Warsaw parks studied here. Soil bulk density differed in relation to the type of forest stands (Table 2). In PR forest stands, the capillary water capacity expressed in weight was lower than in RR forest stands. No such differences were found in volumetric capillary capacity, which indirectly demonstrates the role of organic particles in capillary rising in soils of similar grain size structure in PR forest stands. As was found by Bhuju and Ohsawa (1998), the differences in soil moisture during drought and after rainfall as well as the actual soil moisture and capillary water content between forest stands PR and RR were found to be insignificant (Table 2). The understory vegetation of RR and RO forest stands associated with various habitat continuities differed in physical and chemical soil conditions (with the exception of moist bulk density; Table 2). Hermy (1994) and Verheyen and Hermy (2001) suggested that the habitat conditions and plant density in young forests hamper the penetration of less-competitive species typical of ancient forests. Honnay et al. (1999) concluded that the share of fine-grained soil particles, pH and phosphorus content are also important. However, Graae et al. (2003) were sceptical about soil features such as phosphorus content, organic matter and pH. They demonstrated that these properties were insignificant, and the only factor important for colonisation by forest species was found to be the distance from ancient forests. Under the effects of visitor pressure, the differences became smaller.

No effect of canopy-forming tree species on habitat conditions was found in the studied forest stands. Over a longer time period of several dozen years, it would be expected that the studied trees (Reich et al. 2005) would alter the soil pH, but under the effect of visitor pressure, the influence of dominant tree species appeared to be significant only when interacting with the direction factor (Table 4), which can be related to the users’ preference of shady parts of undergrowth during sunny days. Soil salinity is one of the more important indicators of the human impact on urban green spaces that affect plant growth (Iakovoglou and Thompson 2001; Czerniawska-Kusza et al. 2004). In the studied parks, soil salinity did not exceed 1.5 mS/cm, and it is above this value that the proper development of plant root systems is disturbed (Huinink 1998; Cunningham et al. 2008). However, soil salinity plays an important role in differentiating understory vegetation (Figs. 2 and 3). The higher salinity observed in PR forest stands may be explained by a denser network of park trails, which are also visited in winter and, during this period, are spread with salt. The range of increased soil salinity may reach up to 30 m from trails treated in this manner (Bäckström et al. 2004; Shaw and Reeve 2008). Under natural conditions, the differentiation of micro-habitats is caused by throughflow and stemflow. Characteristic gradients of changing parameters (mainly of pH) are formed with the distance from the tree trunk (Neumeister et al. 1997; Haase et al. 2000). These gradients are more distinct in the case of acid rain (Draaiers et al. 1997). Sarah and Zhevelev (2007) suggested that visitor pressure contributes directly to the homogenisation of both vegetation and habitat conditions at the scale of a single tree crown. Low pH values prevail close to trunks in natural forests due to acid stemflow from trunks during rains and the acid environment of tree roots (Neumeister et al. 1997; Haase et al. 2000). In the examined parks, pH values varied along a single tree canopy gradient depending on the forest stand type. This variability overlaps irregularly with the trampling around tree trunks, which is proved by significant differences in soil compaction and, consequently, vegetation percentage cover, height and its plant composition (Tables 3 and 4). A rather surprising finding of the present study was the results indicating pH differentiation in PR forest stands. Visitor pressure near tree trunks exacerbate already difficult habitat conditions for plants, and decreases in plant height and the number of plant species are most visible there. In such unfavourable conditions for herbaceous plants, tree seedlings seem to find their shelter from trampling (Hauru et al. 2012), which can be an additional factor hampering growth for herbaceous species. A practical recommendation arising from these observations is that areas in which vegetation reacts most intensively to trampling should be treated with special attention (Lucas-Borja et al. 2011). It appears that differentiation of vegetation and habitats does not always indicate improved conservation status. For consistent actions to be undertaken in parks subjected to user impacts as suggested by Bhuju and Ohsawa (1998), it is useful to prepare a recovery plan, which should include restrictions on the movement of visitors and heavy equipment, the elimination of surfaces with destroyed vegetation and, if needed, the implementation of rest periods and management plans. One of the most important and most labile elements in park forest stands are forest species (Hamberg et al. 2008). Therefore, clusters of these species in the understory of ancient forest stands should be totally free from user pressure, especially close to tree trunks.

## Conclusions

Public access to understorey vegetation in forested urban parks results in vegetation impoverishment and a reduction in habitat quality. At the city scale, moderate pressure from park visitors results in a decrease of forest plant species that are rare in the urban environment. In restricted access forests, the percentage of forest species is over 30 times higher than in public access parks. In areas subjected to trampling, native non-forest species prevail, but vegetation percentage cover is lower. The differentiation along a distance gradient around individual trees is manifested in increased soil compaction, pH, moisture difference and soil capillary volume. If restricted access is implemented in urban parks, especially in valuable parts of woodlands with a well-developed understorey vegetation, it could prove to be a valuable method of biodiversity conservation in urban ecosystems. Results from many studies suggest that areas of restricted access should be implemented for better biodiversity preservation, but as the parks’ purpose is to provide multifunctional services there should also be off-trail activities possible to connect people with nature. Proper traffic channelling is the key issue in this manner.

## Appendix 1

^a^—ancient forest species; ^f^—native forest species; ^n^—native non-forest species; ^x^—alien plant species

List of vascular species, abbreviations as in Fig. 4

ace.pla—*Acer platanoides* ^f^ (juv.),

ace.pse—*Acer pseudoplatanus* ^f^ (juv.),

ace.sac—*Acer saccharinum* ^x^ (juv.),

ach.mil—*Achillea millefolium* ^n^,

aeg.pod—*Aegopodium podagraria* ^a,n^,

agr.gig—*Agrostis gigantea* ^n^,

agr.sto—*Agrostis stolonifera* ^n^,

aju.rep—*Ajuga reptans* ^a,f^,

all.pet—*Allairia petiolata* ^n^,

asa.eur—*Asarum europaeum* ^a,f^,

bel.per—*Bellis perennis* ^n^,

cap.bur—*Capsella bursa-pastoris* ^n^,

car.bet—*Carpinus betulus* ^f^,

car.bri—*Carex brizoides* ^f^,

car.pra—*Cardamine pratensis* ^f^,

che.maj—*Chelidonium majus* ^n^,

che.str*—Chenopodium strictum* ^x^,

cor.ave—*Corylus avellana* ^f^,

cor.san—*Cornus sanguinea* ^f^,

cra.mon—*Crataegus monogyna* ^n^,

dac.glo*—Dactylis glomerata* ^n^,

eri.ann—*Erigeron annuus* ^x^,

euo.eur—*Euonymus europaeus* ^f^ (juv.),

fal.dum—*Fallopia dumetorum* ^n^,

fes.rub—*Festuca rubra* ^n^,

fra.exc—*Fraxinus excelsior* ^f^ (juv.),

gal.lut—*Galeobdolon luteum* ^a,f^,

ger.rob—*Geranium robertianum* ^n^,

geu.riv—*Geum rivale* ^n^,

geu.urb—*Geum urbanum* ^a,n^,

gle.hed—*Glechoma hederacea* ^n^,

hed.hel—*Hedera helix* ^a,f^,

hyp.gla—*Hypocheris glabra* ^n^,

imp.par—*Impatients parviflora* ^x^,

lam.pur—*Lamium purpureum* ^x^,

lap.com—*Lapsana communis* ^n^,

lol.per—*Lolium perenne* ^n^,

mai.bif—*Maianthemum bifolium* ^a,f^,

mel.nut—*Melica nutans* ^a,f^,

mer.per—*Mercurialis perennis* ^a,f^,

oxa.ace—*Oxalis acetosella* ^a,f^,

oxa.fon—*Oxalis fontana* ^x^,

pad.avi—*Prunus padus* ^f^ (juv.),

pla.maj—*Plantago major* ^n^,

poa.ang—*Poa angustifolia* ^n^,

poa.ann—*Poa annua* ^n^,

poa.nem—*Poa nemoralis* ^a,f^,

poa.pra—*Poa pratensis* ^n^,

pol.avi—*Polygonum aviculare* ^n^,

pol.mul—*Polygonatum multiflorum* ^a,f^,

pop.alb—*Populus alba* ^f^ (juv.),

pot.rep—*Potentilla reptans* ^n^,

que.rob—*Quercus robur* ^f^ (juv.),

que.rub—*Quercus rubra* ^x^ (juv.),

ran.acr—*Ranunculus acris* ^n^,

ran.rep—*Ranunculus repens* ^n^,

ror.sil—*Rorippa sylvestris* ^n^,

rub.fru—*Rubus fruticosus* agg.^f^,

rum.obt—*Rumex obtusifolius* ^n^,

sam.nig—*Sambucus nigra* ^n^ (juv.),

set.pum—*Setaria pumila* ^x^,

sor.auc—*Sorbus aucuparia* ^f^ (juv.),

sta.syl—*Stachys sylvatica* ^a,f^,

ste.hol—*Stellaria holostea* ^a,f^,

ste.med—*Stellaria media* ^n^,

tar.rud—*Taraxacum* sect. *Ruderalia* ^n^,

til.cor—*Tilia cordata* ^f^ (juv.),

til.pla—*Tilia platyphyllos* ^f^ (juv.),

tri.rep—*Trifolium repens* ^n^,

ulm.gla—*Ulmus glabra* ^f^ (juv.),

urt.dio—*Urtica dioica* ^n^,

ver.ser—*Veronica serphylifolia* ^n^,

vio.odo—*Viola odorata* ^x^,

vio.rei—*Viola reichenbachiana* ^a,f^

## Abbreviations

RO: Old (ancient) forest stand with restricted access to undergrowth vegetation
RR: Recent forest stand with restricted access to undergrowth vegetation
PR: Recent forest stand with public access
